# Disciplinary boundaries and integrating care: using Q-methodology to understand trainee views on being a good doctor

**DOI:** 10.1186/s12909-019-1493-2

**Published:** 2019-02-15

**Authors:** E. Muddiman, A. D. Bullock, J. M. Hampton, L. Allery, J. MacDonald, K. L. Webb, L. Pugsley

**Affiliations:** 1 Cardiff University School of Social Sciences, Cardiff, UK; 2 Cardiff University School of Postgraduate Medical and Dental Education, Cardiff, UK

**Keywords:** Medical generalism, Professional identity, Multidisciplinary, Q methodology, Complex care, Multimorbidity

## Abstract

**Background:**

Rising numbers of patients with multiple-conditions and complex care needs mean that it is increasingly important for doctors from different specialty areas to work together, alongside other members of the multi-disciplinary team, to provide patient centred care. However, intra-professional boundaries and silos within the medical profession may challenge holistic approaches to patient care.

**Methods:**

We used Q methodology to examine how postgraduate trainees (*n* = 38) on a range of different specialty programmes in England and Wales could be grouped based on their rankings of 40 statements about ‘being a good doctor’. Themes covered in the Q-set include: generalism (breadth) and specialism (depth), interdisciplinarity and multidisciplinary team working, patient-centredness, and managing complex care needs.

**Results:**

A by-person factor analysis enabled us to map distinct perspectives within our participant group (P-set). Despite high levels of overall commonality, three groups of trainees emerged, each with a clear perspective on being a good doctor. We describe the first group as ‘generalists’: team-players with a collegial and patient-centred approach to their role. The second group of ‘general specialists’ aspired to be specialists but with a generalist and patient-centred approach to care within their specialty area. Both these two groups can be contrasted to those in the third ‘specialist’ group, who had a more singular focus on how their specialty can help the patient.

**Conclusions:**

Whilst distinct, the priorities and values of trainees in this study share some important aspects. The results of our Q-sort analysis suggest that it may be helpful to understand the relationship between generalism and specialism as less of a dichotomy and more of a continuum that transcends primary and secondary care settings. A nuanced understanding of trainee views on being a good doctor, across different specialties, may help us to bridge gaps and foster interdisciplinary working.

## Background

This paper investigates medical trainees’ views on what ‘being a good doctor’ means in the context of calls for a more generalist approach to caring for patients with complex health needs. Rising numbers of patients with multiple-conditions and complex care needs [1–3] challenge current structures of healthcare organisation when discipline-based specialisation no longer suits their care needs [4]. In particular, ageing populations are driving this change and are shifting conventional demands on health services [4, 5]. Doctors have highlighted concerns about lack of continuity and uncertainty about who has overall responsibility for the care of patients with complex care needs [4]. It has become increasingly important for doctors from different specialty areas to work together, alongside other members of the multi-disciplinary team, to provide patient-centred care [6–11]. The World Health Organisation called for an integrated approach to manage the complex needs of the ageing population [2] and different approaches to care are being tried [4]. The United States (US) employs the term ‘hospitalist’ to describe a generalist physician responsible for patients throughout their hospital stay [4, 11]. Physicians working in Acute Medical Units (AMUs) in the United Kingdom (UK) provide rapid multidisciplinary medical assessment [4]. However, an agreed interpretation of generalism in hospital settings has yet to be established [10]. A 2011 report on *Modern Medical Generalism,* commissioned by the Royal College of General Practitioners and the Health Foundation (UK), warns against a simplistic definition of generalists as providing ‘first-contact’ care, adding that there is more to medical generalism than good practice: ‘although the generic attributes associated with good professional practice are an intrinsic part of generalism, the generalist has specific clinical qualities that go significantly beyond this’. [12] There has been a similar debate within nursing about the relative status of generalists and specialists, with NHS Scotland advocating that specialist nursing to be viewed as being located at ‘one pole of the “specialist generalist” continuum’, and indicating a linguistic shift away from associating ‘specialism’ with ‘seniority’ amongst medical staff [13]. However, our previous research indicates that traditional disciplinary structures within medicine (with the conventional split of generalist practitioners in the primary care or community setting, and specialists based in hospitals) can undermine and complicate trainee generalists’ developing sense of professional identity [14]. In this context, what it means to be a ‘good’ (or even ‘ideal’) doctor [15], in the context of an increasing emphasis on hospital-based generalists that threatens to disrupt traditional (and perhaps outdated) hierarchies of specialism, is a question of growing international concern.

In the UK, *The Shape of Training Review* [7] identified the need to train ‘specialist generalists’ who can offer a more holistic approach to patient care. Broad Based Training (BBT) introduced by Health Education England (HEE) and The Academy of Medical Royal Colleges (AoMRC), was specifically designed to address the generalist agenda [4]. This two-year programme follows the two-year postgraduate foundation training, and provides six-month placements in four specialties (paediatrics, general practice, core medicine, and psychiatry), at the point where postgraduate trainees would normally be entering conventional specialty training. Upon completion, BBT trainees enter onto the second year of specialty training in their chosen specialty. BBT thus extends the overall length of training by one year. By exposing trainees to a breadth of different medical settings and encounters, and by incorporating training techniques specifically intended to foster cross-disciplinary working, BBT aims to develop practitioners adept at managing complex, patient-focussed care and specialty integration.

International literature focused on *patient* perspectives reveals the high importance patients attach to interpersonal qualities (such as communication skills and a caring attitude) over doctors’ knowledge and skills which attracts less emphasis [16–19]. Literature from the Australia [20] and the United States [21] focused on medical *student* opinions also reveals the importance of softer skills (such as empathy or compassion) but in addition gives emphasis to cognitive, clinical and performative skills [15]. Such research amongst patients and students has revealed commonalities and difference of opinion. Research to date suggests that qualities desirable in a doctor include both cognitive and non-cognitive qualities such as integrity, empathy and good people skills. There are also indications that views vary between patients, students and doctors and, over time. Less is known about how views vary between specialty groups. We do not yet know if the professional identity associated with training in some specialties emphasises certain qualities over others or on what challenges generalism poses to existing models of professional identity. This worthy of further research. Clearly the need for a different kind of doctor has implications for training and for subsequent careers guidance and specialty choice. However, what also deserves attention is the challenge from intra-professional boundaries within the medical profession that a more holistic approach to patient care may face [22–25]. A better understanding of the views and perspectives of medical trainees from different specialty areas may help to identify how to bridge disciplinary gaps and foster interdisciplinary work [14, 26].

## Methods

Using Q-methodology [27], we compare medical trainees’ views on what ‘being a good doctor’ means in the context of calls for a more generalist approach to meet increasingly complex patient care needs [5–8]. Specifically, we draw on data collected from postgraduate post-foundation trainees from a range of different medical training programmes in England and Wales, including both conventional specialty training programmes in a variety of medical disciplines, and a ‘broad-based’ programme aimed at fostering generalism and interdisciplinary working. This comparative approach enables us to identify similarities and differences in trainee dispositions according to both onward specialty choice and mode of training (conventional specialised or non-conventional generalised). Research ethical approval was obtained from Cardiff University (02/10/13). Participation was voluntary, informed consent was gained, and participants are anonymised. None of the authors have any competing interests.

### Study context

The research presented here is part of a larger mixed-methods longitudinal study of the BBT programme that was introduced in England in 2013 by the AoMRC and funded by HEE.

The first cohort of BBT trainees (*n* = 42 at outset) were recruited across seven different regions (Local Education and Training Boards – LETBs) in England. Despite successfully recruiting two further cohorts and expanding the scope of the programme to most regions in England in 2015, the decision was made to cease further recruitment in 2016 [28], and the future of the programme remains unclear. Nevertheless, recent pilots in Wales and Northern Ireland and discussions in Scotland indicate that interest in the generalist agenda shows no sign of dissipating [29]. Our research, commissioned by AoMRC and funded by HEE, studies BBT and explores whether it better prepares trainees for onward specialty training and the changing landscape of healthcare delivery. In addition to collecting questionnaire and focus group data at regular intervals [30], we have used Q-methodology to examine trainee perceptions of their future role and ‘being a good doctor’.

### Design

Q methodology allows for the systematic collection of individuals relative opinions towards a phenomena, and is becoming increasingly popular within a range of applied and health related disciplines. Participants are asked to rank a set of statements (or items) relative to one another into a grid according to a scale (e.g. in our study ‘most agree’ to ‘most disagree’), which usually takes the shape of a quasi-normal distribution. Collected together, these completed grids are subject to a dimension reduction technique, sometimes referred to as ‘inverted factor analysis’ [27]. This analysis groups those with similar sorting patterns, indicating similar viewpoints, together. This approach is distinct from most other statistical techniques in which demographic factors, such as gender or age, are employed as controls or used as a basis to sort participants into groups. As Bang and Montgomery [28] (p.346) put it: ‘the aim of Q is to utilize subjectiver views, opinions, and perception to capture general responses to a phenomena’.

### Q-set

The Q-set is a set of statements that the participants sort in order to best represent their perspective. The set aims to capture many (if not all) possible responses to our question of interest: ‘what does it mean to be a good doctor?’. After creating a ‘long-list’, informed by both a review of the literature and focus group data from our wider project [30], members of the research team independently selected a short-list of representative statements, and met to agree a 40 item Q-set. This set was piloted with a separate group of medical trainees (*n* = 11), and two statements were subsequently replaced to make the final Q-set. Themes covered in the Q-set include: generalism (breadth) and specialism (depth), interdisciplinarity and multidisciplinary, team working, patient-centredness, and managing complex care needs (for a full list of statements see the first column of Table 4).

### Study participants and data collection procedures

Data were collected from BBT trainees at a national meeting in London (May 2015) and from additional groups of BBT and ‘conventional’ trainees at various regional learning events (April–August 2015). We report Q-sort data from postgraduate trainees (*n* = 38) from a range of different specialty programmes on either the BBT (*n* = 16) or conventional specialty training programmes (*n* = 22) (see Table 1). This sample size was deemed appropriate following an adaptation of Kline’s [31] advice for ‘traditional’ factor analysis, alongside Stainton Rogers’ [32] advice for Q studies. All participants from the BBT programme were in the second year of their programme and were preparing for the transition into their chosen onward specialty (general practice *n* = 8, paediatrics *n* = 5, medicine *n* = 2, psychiatry *n* = 1). Those in our ‘conventional’ group were already focussing solely on their career specialty but had different levels of experience/seniority. We initially sought participants on conventional training courses in one of the four BBT specialty areas, but later decided to include trainees in different hospital-based specialty areas (including general surgery, oncology and gastroenterology) to increase our sample size and maximise potential variability [27].

**Table 1:** Participants in our 2015 Q-sort exercise (P-set)

Specialty		Number
BBT		16
Medicine		10
General Practice		2
Paediatrics		2
Psychiatry		1
Other hospital-based specialisms		7
Total		38

**Fig. 1:**
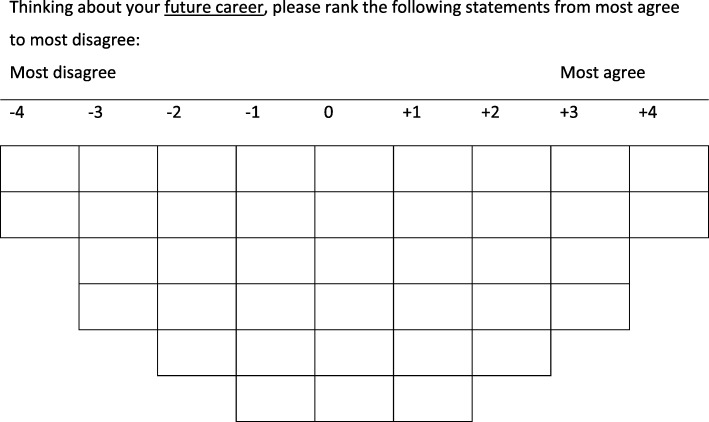
The Q-sorting grid

Participants were asked to first sort the statements into three piles to represent whether they agreed, disagreed or were unsure/neutral that the statement represented a good doctor in terms of their future career. As well as enabling the participants to gain some familiarity with each statement, the number of statements in each pile was recorded in order for the researchers to understand participants’ *overall* level of agreeability towards the statements. Participants were then directed to distribute each pile of statements onto the Q-sort grid along a continuum from most disagree to most agree. As is common in Q-sort studies, our grid had a forced, quasi-normal distribution (see Fig. 1). The placement of individual statements on this grid creates an overall sorting pattern unique to the participant. This pattern can then be examined holistically in relation to other participants’ sorting patterns. Once the Q-sorting process was complete and recorded, participants filled out a post-sort questionnaire that invited comment on their sorting choices.

### Data analysis

The completed Q-sorts from all participants were analysed using PQMethod 2.35 [33]. The software allows inverted or by-person factor analysis to be conducted, which identifies similarities (or shared variance) between the sorting patterns of respondents. Various extraction and rotation techniques are possible, we chose to follow traditional Q methodology studies (such as those by Stephenson [34] and, latterly, Brown [35]) and use centroid extraction. Rotation allows for the factors to become more easily interpretable and is commonly used in almost all dimension reduction analyses. In this case, a varimax rotation was used. This process of rotation, identification and extraction generates a number of factors – grouping participants with similar responses together. Following best practice, we only considered factor groupings, or ‘solutions’ with Eigenvalues > 1, and with at least two statistically significant participant Q-sorts loaded onto each factor [27]. This left us with five viable solutions comprising of two to six factors. The research team then had some analytical discretion when deciding which ‘solution’ or number of factor groups, best represents the data. Following the maxim that the most appropriate solution is that which offers a ‘simple solution’ [36] - describing the structure of the data whilst simultaneously providing a readily interpretable substantive solution – the team agreed that a three-factor solution met all these criteria and offered the most explanatory power.

## Results

Our three factor varimax solution explains 61% of total study variance and accounts for 27/38 participants at a 0.50 significance level (see Tables 2 and 3).

**Table 2:** Factor (group) loadings according to specialty

Specialty	# Participants	# Factor loadings
BBT	16	8
General Practice	2	2
Medicine	10	7
Paediatrics	2	2
Psychiatry	1	1
Other hospital-based specialties	7	6
Total	36	27

**Table 3:** Factor eigenvalues and percentage variance explained

Factor group	Eigenvalue	% variance explained
A	7.68	20
B	9.54	25
C	5.09	13

From the factor arrays (see Table 4), we compiled a ‘sorting pattern’ for each group, highlighting the statements ranked higher or lower than each of the other groups (not including tied rankings). Those statements identified by PQMethod as consensus statements were not included in these lists (except when they have been placed in the ‘top’ or ‘bottom’ position) as it has been established that they do not differentiate the groups from one another. Members of the research team used these contrasting sorting patterns [37], in conjunction with the overall factor arrays and participants’ post-sort questionnaire responses, to interpret participants’ collective subjective viewpoints on what ‘being a good doctor’ means [38]. This holistic approach to interpretation is common in Q methodology, with results of the factor analysis often aided by theory and/or cultural knowledge [39].

**Table 4:** Factor arrays^a^

#	Statement	Factor A	Factor B	Factor C
1	Having a particular mind-set.	−3	− 3	−3
2	Focussing on how my specialty can help the patient.	0	0	3
3	Keeping abreast of medical developments across different related specialties.	0	0	1
4	Having a depth of medical knowledge in my specialty.	0	3	0
5	Having a breadth of medical knowledge.	4	1	1
6	Paying attention to the overall wellbeing of individuals.	3	4	0
7	Bending the rules when necessary.	−1	−2	−3
8	Mastering specific skills.	−3	2	2
9	Knowing how to care for patients with complex care needs.	2	2	1
10	Consulting with others when I don’t have the answer.	1	3	3
11	Making appropriate referrals.	2	0	−1
12	Making clinical decisions on a case-by-case basis.	2	2	1
13	Empowering patients to make decisions regarding their treatment.	1	1	1
14	Acting on the individual needs of my patients.	2	3	2
15	Understanding the community to which my patient belongs.	1	0	−2
16	Adapting to changes in the NHS.	0	−1	−1
17	Orchestrating care for patients with multiple conditions.	3	1	−2
18	Re-training if necessary to match service demand.	−3	−2	−3
19	Understanding the links between specialties.	−1	0	−1
20	Reaching consultant/partner status.	−2	−3	− 2
21	Understanding the limitations faced by those working in other specialties.	−1	−1	−2
22	Being open to ideas about doing things differently.	0	1	2
23	Focussing exclusively on my specialty at an early stage in my training.	−3	−3	−4
24	Thinking about the overlaps between different specialties.	−2	−1	0
25	Understanding how medical conditions outside my own specialty impact on my patients.	−2	1	0
26	Being able to deal with diagnostic uncertainty.	3	0	3
27	Being able to isolate the issue at hand.	1	−1	0
28	Taking the lead.	0	−1	−1
29	Having a good work-life balance.	1	−2	4
30	Acting confidently even when I am unsure.	−1	−3	−3
31	Being well remunerated.	−1	−4	−1
32	Being respected by others.	−2	−2	0
33	Having the final say in the multidisciplinary team.	−4	−4	− 4
34	Acknowledging the expertise of others in the multidisciplinary team.	1	2	2
35	Being an expert.	−4	−1	−1
36	Prioritising my NHS practice over private work.	−2	−2	−2
37	Having excellent communication skills.	4	4	3
38	Acting with compassion.	3	3	4
39	Constantly challenging myself.	−1	1	2
40	Attending to the emotional aspects of my patients’ experiences.	2	2	1

^a^The scores in the factor columns correspond to the ranked placement of the statement for each group, from most disagree (−4) to most agree (4)

### Overall commonality, differentiating statements, and agreeability

Firstly, it is notable that there is a high degree of commonality across all three groups (inter-correlated across the board at 0.67). This means that whilst some statements were placed differently by those in different factor groups, almost half (*n* = 18) were positioned in a very similar fashion by *all* participants regardless of their factor grouping (consensus statements are italicised in Table 4). These statements might therefore be regarded as universal across the participant groups. For example all participants ranked ‘having excellent communication skills’ and ‘acting with compassion’ very highly. These priorities were reflected in participants’ post-sort comments:

A doctor without compassion has no place in the medical profession! (Conv.37)

Communication is key to ensuring patient understanding and helping them to take charge of their own health (BBT.14)

Without the ability to communicate why a decision has been reached patients may be confused and angry (Conv.27)

At the opposite end of the scale all participants ranked ‘having the final say in the multidisciplinary team’ lowest (most disagree) and also ranked ‘having a particular mindset’ and focussing exclusively on my specialty at an early stage in my training’ relatively low:

The whole point of the multidisciplinary team is collaborative working and no member should have a louder voice than another. (Conv.24)

Early training should have a general approach as most patients have multiple issues alongside their specialty specific ones (Conv.29)

Having a fixed mind-set can lead to missing information/poor care (Conv.23)

The overall level of commonality in our P-set means that there was only one statistically significant distinguishing statement. The statement ‘mastering specific skills’ distinguishes group A from all other group (A = -3, B = + 2, C = + 2). This means that individuals in group A ranked this statement significantly lower than those in the other two factor groups.

On average, participants tended to ‘agree’ with over half of the statements, suggesting high overall agreeability. This is helpful when interpreting the Q-sorts because it indicates that items placed towards the left-hand side of the sorting grid may not indicate that participants have ‘disagreed’ with them, just that they agree with them *less* than all of the statements further to the right of the grid. BBT trainees had a slightly higher average agreeability rating (27.0) compared to those on conventional programmes (23.7), indicating that BBT trainees tended to agree with more statements overall.

### Three factor groups

Despite high levels of overall commonality, three groups of trainees emerged, each with a distinct perspective on being a good doctor. The distinctiveness of the factors is made apparent in the following, which explores the subtle but important differences in voice and opinion within the overall participant group. The three factor groups are examined in turn, using characterising statements taken from the factor arrays, along with explanations from the post-sort questionnaires. The characteristics of those loading onto the groups are also raised where salient to interpretation but are not tested for statistical difference between factors given the probabilistic assignment of loadings. The names given to the factors reflect the generalist-specialist continuum which emerged from the holistic, substantive interpretation process.

### Group a: Generalists

This group gave highest priority to ‘having excellent communication skills’ and a ‘breadth of medical knowledge’. They also emphasized ‘understanding the community to which my patients belong’, ‘orchestrating care for patients with multiple conditions’ and ‘making appropriate referrals’. These priorities were reflected in comments made in their post-sort questionnaires:

Being a GP (eventually) I envisage needing a wide range of knowledge in various specialties as patients will be consulting me about so many things (BBT.03)

It is important to be able to adapt communication styles to the needs of the patient and make them feel at ease during the consultation (BBT.08)

They gave lowest priority to ‘being an expert’ and ‘having the final say in the multidisciplinary team’. The overall positioning of statements of those in this group, coupled with their post-sort questionnaire comments led us to interpret them as generalists and team-players with a collegial and patient-centred approach to their role. This orientation to medicine was particularly popular amongst those on conventional GP training programmes and those opting to follow GP pathways post-BBT:

I believe being a good GP entails well-rounded, emotionally-balanced individuals; involved in holistic care for their patients; with a keen sense of their role and responsibility in the healthcare system (BBT.12)

GPs should be holistic practitioners who care about their patients’ mental and physical health and are aware of the effects that social problems can have on health. They should understand the communities they work in (Conv.36)

Of participants with significantly loading Q-sorts, this group (*n* = 8) contains *all* of those aspiring to be GPs from both BBT and conventional GP training (*n* = 6, 100%), and 22% of trainees on conventional training pathways in medicine (*n* = 2). A higher proportion of those on the BBT programme (50% of BBT trainees with significantly loading Q-sorts) are in this group, compared with 21% of trainees on conventional programmes. It also represents 33% of male participants and 28% of female participants.

### Group B: General-specialists

This group focussed on ‘paying attention to the overall wellbeing of individuals’ and ‘having excellent communication skills’. Whilst they prioritised ‘having a depth of medical knowledge’, they also wanted to understand the ‘links between specialties’:

It is important not to ignore other aspects of patient care to focus entirely on a specialty (Conv.27)

Focussing on wellbeing rather than just treating disease gives more holistic care and wider benefits (BBT.02)

They ranked ‘reaching consultant status’ and ‘being well remunerated’ lower than those in other groups. The overall configuration of this group alongside their post-sort questionnaires suggest that they aspire to be specialists but with a generalist and patient-centred approach to care within their specialty area:

Being a good doctor means having a great deal of expertise in a specialist area, taking the patient as the centre of care but remaining open to new ideas and recognising the importance of other specialties and health professionals contributing to patient care (Conv.27)

This orientation to medical care was seen to resonate with those pursuing a career in Paediatrics:

A specialist in children but a generalist with a holistic outlook to coordinate complex cases with lots of MDT and inter-specialty working (Conv.38)

Of participants with significantly loading Q-sorts, this group (*n* = 12) includes *all* those pursuing a career in paediatrics (on both conventional and BBT training), and five from the nine (55%) participants in conventional training in medicine (including gastroenterology and those with an interest in oncology and elderly care as sub-specialties). Three BBT trainees (38% of those with significantly loading Q-sorts) are in this group, compared with almost half 47% of trainees on conventional programmes. Thirty-three percent of male participants and 50% of female participants with significantly loading Q-sorts are in this group.

### Group C: Specialists

This group placed ‘having a good work-life balance’ and ‘acting with compassion’ as their highest priorities. They focussed on how their specialty could help the patient, constantly challenge themselves, and to be respected by others.

I really value time spent outside of work, and want work and life to complement each other rather than be imbalanced (B.05)

They placed less emphasis on making appropriate referrals, paying attention to the overall wellbeing and emotional aspects of patients’ experiences, and orchestrating care for multiple conditions. Our analysis of this group suggests that they aspire to being highly specialised and progressing in their own medical career. These priorities are summed up in these participants’ responses to the post-sort question ‘what is your overall view on what being a good doctor means?’ which focus on individual competence and dynamism:

Competent, able, knowledgeable (Conv.20)

Good level of knowledge and practical ability. Able to react competently to rapidly changing acute situations (Conv.35)

Of the participants with significantly loading Q-sorts, this group (*n* = 7) includes just one BBT trainee (pursuing onward training in medicine), and six trainees on conventional training programmes (32% of those with significantly loading Q-sorts). Two of the nine (22%) trainees pursuing careers in medicine (including gastroenterology) can be found in this group, along with the *only* psychiatry trainee, and four of the six (66%) trainees in other hospital-based specialties (not including medicine, paediatrics or psychiatry). One third of male participants and 22% of female participants with significantly loading Q-sorts are in this group.

## Discussion

Group A, labelled by us as ‘generalists’ seem to be most aligned to the generalist agenda outlined by Greenaway [7] amongst others. It is interesting that a higher proportion of BBT trainees than those on conventional training courses fall into this category. This group is dominated by those aspiring to be GPs in the primary care setting, but also those training in core medicine who are likely to be hospital-based. The second group of ‘general-specialists’ (group B), was dominated by those aspiring to be paediatricians, suggesting that those in secondary care focussing on a patient group (e.g. children/the elderly) may have more generalist outlooks than those focussing on a particular body part or system. Both these two groups can be contrasted to those in the third ‘specialist’ group (C), who seem to have a more singular focus on how their specialty can help the patient. Members of this group from ‘other specialties’ include general surgery, neurology and histopathology.

### Do trainee perspectives vary according to specialty?

For those participants following the novel BBT programme, each group appears to represent a particular specialty area: of those significantly loading onto a factor, all those aspiring to be GPs can be found in group A, all those aspiring to be paediatricians are located in group B and the one trainee in group C is pursuing an onward career in medicine. This suggests that these trainees’ onward career specialty aspirations distinguish them from one another. However, it is important to note that two thirds (*n* = 6) of those BBT trainees who *did not* load significantly onto a single factor (*n* = 8) were confounded between groups A and B, suggesting that the overwhelming majority of BBT trainees self-identify as generalists or general specialists (as opposed to specialists).

The picture is less clear, however, for those on conventional specialty training programmes. On the one hand, following the pattern of the BBT trainees, all those training to be GPs can be found in group A and all those training to be paediatricians can be found in group B. However, on the other, those pursuing a career in Medicine (broadly defined) can be found across *all three* groups, and those in ‘other’ hospital-based specialties not including medicine, paediatrics, and psychiatry are located in groups B and C. This may be due to variation in sub-specialty roles within some broad specialty areas and could be a reflection of the wider sampling of those on conventional pathways compared to the BBT group.

### Do trainee perspectives vary according to gender?

Of those with significantly loading Q-sorts, males are spread evenly between the three groups, whilst half of female participants fall into group B, with 28 and 22% in groups A and C respectively. This is perhaps unsurprising given that group B includes all of those participants with significant loadings pursuing careers in Paediatrics and it has been shown that women make up over two-thirds of Paediatric trainees in England [40].

### Do disciplinary boundaries mask underlying commonalities?

We have noted the high levels of overall commonality between participants’ Q-sort configurations, indicating that trainees across different specialty areas share similar views on a number of key issues. These data therefore suggest that inter-disciplinary boundaries and the assumption that those in different medical specialty areas think and act differently, may mask underlying commonalities and universal values held by trainees and shared understandings about what ‘being a good doctor’ means across interdisciplinary divides.

### Limitations and future directions

Whilst the correlations that form part of the inverted factor analysis characteristic of Q-sort analysis mean that the numbers of participants required is typically few than the number of statements (here 40) [27], it is regrettable that we were not able to include more BBT trainees, or those specialising in Psychiatry in our sample. Another potential sampling issue is that our participants came from different stages in their postgraduate training specialties. However, rather than trying to control for differences such as this, proponents of Q-methodology encourage sampling for maximum diversity in order that the by-person factor analysis *reveals* which demographic or other characteristics are pertinent to understanding the grouping of individual Q-sorts. We did not find stage of training to be an important factor in our analysis of our three groups. Another potential limitation of studies using Q-methodology is that the concourse or Q-set can never be exhaustive and a balance must be achieved between overall number of statements and the manageability of the task. By piloting our Q-set and asking participants to indicate if they would add any statements in their post-sort questionnaire we have sought to minimise the impact of this limitation.

Building on this exploratory study of trainee’ doctors perspectives in relation to key debates on generalism and specialism, more research into both the similarities and differences in outlook of doctors in different disciplines is needed in order to appreciate more fully the relationship between inter- and intra- professional identities [22]. Indeed, by casting our net of participants widely, our analysis may have obscured subtler differences within disciplinary groups. A fruitful avenue for further research may be, therefore, to explore perspectives according to stage of training, gender, and within specialist groupings.

## Conclusions

There is growing international recognition of the potential for integrated care and enhanced medical generalism [6–8, 10–12] to alleviate the challenges presented by ageing populations and the associated rise in complex care needs and co- and multi-morbidities [1–3, 7–9]. This project demonstrates the potential for Q-methodology to address research questions relating to the perspectives and subjectivities of healthcare professionals in a systematic and ‘qualiquantological’ [41] manner. The results of our Q-sort analysis suggest that it may be helpful to understand the relationship between generalism and specialism as less of a dichotomy and more of a continuum that transcends primary and secondary care settings. This will have implications for medical education and training. A nuanced understanding of trainee views on being a good doctor, across different specialties, may help us to bridge gaps and foster interdisciplinary working.

## Abbreviations

AMU: Acute Medical Unit
AoMRC: Academy of Medical Royal Colleges
BBT: Broad Based Training
GP: General Practitioner
HEE: Health Education England
MDT: Multidisciplinary Team
